# Using Wearable Devices and Speech Data for Personalized Machine Learning in Early Detection of Mental Disorders: Protocol for a Participatory Research Study

**DOI:** 10.2196/48210

**Published:** 2023-11-13

**Authors:** Ramon E Diaz-Ramos, Isabella Noriega, Luis A Trejo, Eleni Stroulia, Bo Cao

**Affiliations:** 1 Department of Computing Science University of Alberta Edmonton, AB Canada; 2 School of Engineering and Sciences Tecnologico de Monterrey Monterrey Mexico; 3 School of Engineering and Sciences Tecnologico de Monterrey Atizapan Mexico; 4 Department of Psychiatry University of Alberta Edmonton, AB Canada

**Keywords:** machine learning, speech analysis, Depression, Anxiety, and Stress Scale, DASS21, depression, anxiety, stress, mood disorders, mental health, voice, smartwatches, wearables

## Abstract

**Background:**

Early identification of mental disorder symptoms is crucial for timely treatment and reduction of recurring symptoms and disabilities. A tool to help individuals recognize warning signs is important. We posit that such a tool would have to rely on longitudinal analysis of patterns and trends in the individual’s daily activities and mood, which can now be captured through data from wearable activity trackers, speech recordings from mobile devices, and the individual’s own description of their mental state. In this paper, we describe such a tool developed by our team to detect early signs of depression, anxiety, and stress.

**Objective:**

This study aims to examine three questions about the effectiveness of machine learning models constructed based on multimodal data from wearables, speech, and self-reports: (1) How does speech about issues of personal context differ from speech while reading a neutral text, what type of speech data are more helpful in detecting mental health indicators, and how is the quality of the machine learning models influenced by multilanguage data? (2) Does accuracy improve with longitudinal data collection and how, and what are the most important features? and (3) How do personalized machine learning models compare against population-level models?

**Methods:**

We collect longitudinal data to aid machine learning in accurately identifying patterns of mental disorder symptoms. We developed an app that collects voice, physiological, and activity data. Physiological and activity data are provided by a variety of off-the-shelf fitness trackers, that record steps, active minutes, duration of sleeping stages (rapid eye movement, deep, and light sleep), calories consumed, distance walked, heart rate, and speed. We also collect voice recordings of users reading specific texts and answering open-ended questions chosen randomly from a set of questions without repetition. Finally, the app collects users’ answers to the Depression, Anxiety, and Stress Scale. The collected data from wearable devices and voice recordings will be used to train machine learning models to predict the levels of anxiety, stress, and depression in participants.

**Results:**

The study is ongoing, and data collection will be completed by November 2023. We expect to recruit at least 50 participants attending 2 major universities (in Canada and Mexico) fluent in English or Spanish. The study will include participants aged between 18 and 35 years, with no communication disorders, acute neurological diseases, or history of brain damage. Data collection complied with ethical and privacy requirements.

**Conclusions:**

The study aims to advance personalized machine learning for mental health; generate a data set to predict Depression, Anxiety, and Stress Scale results; and deploy a framework for early detection of depression, anxiety, and stress. Our long-term goal is to develop a noninvasive and objective method for collecting mental health data and promptly detecting mental disorder symptoms.

**International Registered Report Identifier (IRRID):**

DERR1-10.2196/48210

## Introduction

### Overview

The Quantified Self movement proposes that self-tracking, measurement, and quantification of one’s daily life using technology can lead to personal insights that can help individuals improve their behaviors and health. Recent advances in sensors and their embedding in many wearables and mobile devices have made this idea extremely easy to adopt [1]. Currently, affordable consumer devices contain sensors that continuously and unobtrusively track one’s activity, sleep, heart rate, and stress levels. Furthermore, many mobile apps enable individuals to record multimedia throughout their day, such as media, images, audio, and video. Hundreds of MBs of data can be collected from an individual every day [2], thus creating a data stream amenable to machine learning algorithms that can capture patterns related to different behavioral, cognitive, and mental states. In this project, we investigate the usefulness of activity trackers and voice recordings as data sources through which models of mental health indicators can be extracted. More specifically, we are interested in examining how personalized models, constructed from long-term individual data streams, compared with population-level models, learned from population-level data in terms of their ability to predict mental health conditions accurately.

We developed a special-purpose app to collect voice, physiological, and activity data for this study. Physiological and activity data are provided by various off-the-shelf fitness trackers, which record steps, active minutes, duration of sleeping stages (rapid eye movement, deep, and light sleep), calories consumed, distance walked, heart rate, and speed. In addition, our study app collects 2 types of voice recordings: the former is a recording of the user reading a specific text, and the latter is a recording of the user responding to an open prompting question, chosen randomly from a set of open questions, without repetition. Finally, the app collects the users’ answers to a standard psychological instrument, the Depression, Anxiety, and Stress Scale (DASS21) [3], which quantifies the severity of these 3 mental disorders based on the emotional states during the past week, and the results allow us to set the dependent variable of the machine learning algorithm for its classification.

The data collected through our study have already been used for detecting depression, anxiety, and stress [4]. Smartwatch activity data [5-8] and voice [9-11] have been demonstrated to predict depression, anxiety, and stress with high levels of accuracy. The novelty of our study lies in three pillars:

1. First, our study is designed to collect longitudinal data from each participant. In contrast, previous studies about detecting depression from voice relied on data sets consisting of a single data point per participant for a one-size-fits-all model. In addition, each data point includes 2 different voice samples, 1 text reading and 1 personal response to a prompt. This design enables us to analyze the complete data set to train the classifiers of the DASS21 scores and construct personalized models from each participant’s data.
2. Second, besides voice data, our study includes physiological and activity data collected by any number of off-the-shelf fitness trackers, relying on the core subset of common data collected by most such devices today. So far, no study has fused these 2 types of data streams.
3. Third, we are recruiting both English-speaking and Spanish-speaking participants because we aim to develop a language-agnostic, data processing and analysis pipeline.

This paper aims to describe the design of the experiment to capture data from participants and define the study’s objectives for analysis to advance research in mental health disorder detection and personalized machine learning in computing science.

### Background

Personalized machine learning involves using algorithms trained on user data to automatically learn models and use this knowledge to make predictions from new data. Unlike traditional machine learning methods, the results of these models are tailored to an individual and not to a population [12].

Applications of personalized machine learning include heart rate profiling [13], personalized sleep monitoring [14], personalized chronotherapy [13], and studies that predict risk factors for a particular illness [15,16]. A similarity among the previous applications is that the studies have been longitudinal. Longitudinal studies are particularly useful in personalized machine learning, as they permit researchers to track individual data over time and gather data from various factors affecting the changes observed in the results. In these studies, multidimensional data are collected from individuals, and a model is created for each individual.

Typically, models are constructed for predicting one among a set of alternative future states of interest using a classification technique. For example, Feng et al [17] used binary classification to predict whether a specific drug combination would result in a therapeutic response. Webb et al [18] analyzed data from individuals with major depressive disorders and built a model to predict their likelihood of response to antidepressants. Regression models may be developed to forecast a variable’s specific interest value. For example, Bertsimas et al [19] used a k-nearest neighbors regression model to predict blood sugar levels based on individual data from patients with type 2 diabetes. By leveraging the user’s features, the learning algorithms capture specific behaviors of the individual, and their performance is optimized for that particular person.

Early indicators of mental health disorders could motivate early treatment, potentially preventing more severe symptoms that could affect the quality of life of an individual. Preventing these diseases could reduce the symptoms’ recurrence and associated disabilities [20]. These indicators could motivate people to seek the advice of a health professional and receive a prompt and correct diagnosis that could prevent severe depression symptoms in the future. Symptoms are usually diagnosed based on the patient’s description and mental state. Hence, creating a tool to detect early signs of mental diseases is essential to take the necessary measures to prevent major onsets of these diseases.

Therefore, one must address two key questions: (1) *What types of data convey information relevant to extracting indicators of mental-health conditions?* and (2) *How can one collect ground truth on whether the individual is experiencing mental-health challenges?* There is substantial evidence to support the idea that the onset of mental health disorders often coincides with changes in the individual’s daily activity patterns [21]. This information can be realistically captured by the data collected through activity trackers. Although significant studies have been conducted about extracting mental health indicators from activity trackers [4,8], our study aims to use this information without considering the brand, as we strive to compare traditional machine learning methods with personalized modeling. This approach may allow more widespread and accessible data collection, providing more comprehensive insights into mental health. Furthermore, recent studies have established that sound features and content from speech capture relevant information that can be exploited to recognize mental health conditions. This is why our study envisions a regular collection of 2 types of voice recordings: one recording of the users reading a standard text and a second recording of the user responding to a prompting question. To collect the ground truth about our participants’ mental health status, we used the DASS21 instrument, validated for depression, anxiety, and stress [22]. Finally, recognizing that activity data and voice features may exhibit different patterns across individuals, our study is designed to construct personalized models in addition to cohort-based models. We anticipate that these personalized models could improve the accuracy of detecting mental health disorders.

### Related Studies

Machine learning algorithms have been increasingly used for detecting depression, anxiety, and stress in recent years. Several studies have explored machine learning techniques, including deep learning and parametric and nonparametric techniques, to identify patterns in data from physiological measures, self-reported symptoms, and social media posts [23-25]. These studies have shown promising results in accurately predicting mental health outcomes, indicating the potential for machine learning to improve the detection and management of depression, anxiety, and stress.

Personalized machine learning has been increasingly used in health care to provide tailored interventions to individuals based on their unique characteristics and health status. Several studies have demonstrated the potential of personalized machine learning in improving health outcomes, such as predicting patient outcomes, personalized treatment recommendations, and identifying at-risk populations [26,27]. In mental health care, customized machine learning has been used to identify individuals at high risk for suicide and to provide personalized interventions based on individual characteristics and needs [28].

Wearable devices, such as smartwatches and fitness trackers, have emerged as a promising tool for detecting mental health conditions. Several studies have investigated wearables data in combination with machine learning algorithms to detect various mental health conditions, such as depression, anxiety, and bipolar disorder, by analyzing physiological data, such as heart rate and sleep patterns [29,30]. These studies have shown that wearables can provide a discreet and objective way to monitor mental health, allowing for early detection and personalized treatment. However, most studies are performed in clinical setups or with specific features found only with unique equipment. We address this issue by acquiring the core subset of common features among 3 major brands (Fitbit, Samsung, and Garmin) of fitness trackers for framework flexibility.

Using voice and machine learning to detect mental health issues is an active research area. Dematties et al [31] used speech features extracted from recorded interviews to distinguish between individuals with and those without major depressive disorder. Chen et al [32] analyzed the speech of patients with schizophrenia and found that certain speech features indicated the severity of their symptoms. Wang et al [33] used a machine learning algorithm to predict depression and anxiety from speech data obtained during therapy sessions. These studies suggest that voice and machine learning can potentially be valuable tools for the early detection and management of mental health issues. However, the analyzed data consist of a single data point per participant and language. Thus, the resulting data set could provide great insight into multilingual machine learning models and a deep analysis of mood over time.

### Hypothesis and Research Questions

Our study is designed to investigate the following hypothesis: basic activity tracking, in combination with regularly and consistently captured speech data, can provide the basis for extracting accurate indicators of several prevalent mental health conditions, in this case, depression, anxiety, and stress.

Our focus is to comprehend the relative usefulness of the 2 data streams. We aim to investigate the significance of speech data when the user is reading a neutral text versus reflecting about their daily life experiences. In addition, we aim to compare the accuracy of personalized machine learning models with population-level models and evaluate the robustness of these models across various languages.

Our study will answer the following research questions:

1. How does voice modality affect predictive machine learning models, specifically comparing the impact of voice from a neutral text reading against that of voice from open questions about life experiences?
2. What critical predictive features exhibit strong association with DASS21 responses from participants?
3. To what extent does personalized machine learning demonstrate superior predictive power in mental health compared with traditional, one-size-fits-all models in the context of DASS21 responses?

## Methods

### Study Overview and Design

The study analyzes and applies machine learning models to behavioral data extracted from sensors embedded in wearable devices and voice to predict DASS21 results in participants. The data consist of two principal components: (1) sensors embedded in standard smartwatch devices (brands: Samsung, Fitbit, and Garmin) and (2) voice recordings.

Modern sensing technologies in wearable devices continuously measure the body’s autonomic response. These responses include continuous heart rate, daily steps, speed, burned calories, and sleeping patterns (minutes in rapid eye movement sleep, deep sleep, and light sleep) and are monitored daily for the duration of the study. The voice data are collected using 2 methods: the first one is a text reading, which stays consistent with all participants, and the second one is a free-form speech (1 open question is asked). For 2 months, every third day, the participant submits voice samples and DASS21 answers.

The data will show variational patterns that could explain the DASS21 results, and machine learning algorithms can capture these consistent patterns that allow us to predict depression, anxiety, and stress.

### Data Collection

#### Overview

Data collection for this study is ongoing. We expect to collect multidimensional data from at least 50 participants attending 2 major universities—the University of Alberta and Tecnológico de Monterrey, the former from the campus in Edmonton, Alberta, Canada, and the latter from 2 campuses in Mexico, Monterrey and State of Mexico campuses.

The selection criteria considered students aged between 18 and 35 years, with no chronic physical illnesses, high levels of chronic pain, or motor disability and who confirmed not being pregnant. Given that informed consent was required, the study excluded individuals with communication disorders, acute neurological diseases, or a history of brain damage.

Each participant contributes data over 2 months. Participants are assigned an alias as an identification number and are asked to download a mobile app on their mobile phones. The app was developed by the researchers in this study and included the mechanisms to collect the voice recordings and DASS21 questionnaires. Once collected, all data are stored in a repository inside the servers of the University of Alberta associated with this ID. Activity data are collected using Fitbit, Samsung, and Garmin smartwatches, and the app was made available only for Android mobile devices to collect voice and DASS21 answers and upload the smartwatch data.

After this initial assessment, the app regularly (every 3 d) prompts the participants to perform the following tasks. We have chosen 3 days as the app use period to collect samples on different days of the week; this could aid in reflecting the effect of day-to-day stress and workload.

#### Guided Reading

The participants are prompted to read out a paragraph. This task aims to capture as much phonetic variety as possible. For this task, we considered using established English and Spanish text snippets for speech analysis, such as the Calling Stella passage [34].

#### Free-Form Speech

In this task, the participants are asked 2 questions, randomly selected from a list of questions, to answer freely. This includes describing their hobby, city, favorite person, or memorable event. In addition to intonation, these data will enable us to analyze speech-to-pause ratio and durational features, which may correlate with their psychological condition.

#### Smartwatch Data

The participant downloads the data from the smartwatch (we provide the instructions depending on the model and brand) and then uploads the data to the app. The number of attributes and amount of data varies depending on the model and brand of the participant’s smartwatch. However, we have identified and selected common base attributes of each smartwatch model.

Finally, once the abovementioned recordings are complete, participants are prompted to complete a mental health questionnaire (DASS21). The questionnaire gives us a valid indicator of their anxiety, depression, and stress levels to label the audio recordings.

The app lists the mental health resources available to the students of the University of Alberta and encourages them to seek help if they feel stressed, anxious, or depressed. For participants of Tecnológico de Monterrey, it provides a list of mental health resources available at their campus in Mexico.

### Ethical Considerations

The protocol was reviewed and approved by the Research Ethics Office of the University of Alberta (Pro00116909). All participants provided written informed consent after being briefed about the study’s goals. The data are collected using secure transfer mechanisms to comply with ethical and personal data privacy requirements.

### Data Analysis

#### Overview

One of the first steps in data analysis is data exploration. Exploratory analysis is a crucial step in data science, as it helps uncover insights and patterns in the data that can inform subsequent analyses and models. It involves summarizing and visualizing the data, identifying relationships between variables, detecting outliers and missing values, and checking for data quality issues. It also helps to identify potential biases or confounding factors that may influence the results of subsequent analyses.

The purpose of our investigation is to address the abovementioned research inquiries. This section presents our analysis and approaches to examine each question.

#### Question 1

What is the effect of voice modality on predictive machine learning models, specifically comparing the impact of voice from a neutral text reading against that of voice while answering open questions about life experiences?

This question aims to perform a comparative analysis between 2 different voice data sets. These data sets include 2 recordings taken every third day and the answers to the DASS21 questionnaire. The DASS21 questionnaire result is the independent variable we want to predict. DASS21 adopts a dimensional perspective of a psychological disorder rather than a definite one, and it is designed to measure the severity of depression, anxiety, and stress. The scale consists of a numerical value ranging from 0 to 63. The higher the value, the higher the severity of depression, anxiety, and stress.

For this objective, we aim to train 2 machine learning models: one containing the data of a neutral text reading and the other with the voice while answering open questions. We must extract numerical features that quantitatively represent the audio to train the models. These features include but are not limited to language-agnostic linguistic features (eg, number of words, words/min, lemma/token ratio, and verbs/utterance) and perceptual features such as loudness, brightness, pitch, timbre, and rhythm. We will use ensemble methods, such as random forest and support vector machine for the machine learning algorithms that have been demonstrated to have good performance to detect mood disorders using these features [10], and deep learning methods, such as convolutional neural networks and recurrent neural networks [10,35]. We will test both approaches of machine learning tasks, binary classification and regression. For the classification task, the cutoff points are defined based on the DASS21 scale. All scores above mild scores are considered to be indicative of depression, anxiety, or stress for the classification task.

To validate our results, we will use leave-one-out, stratified cross-validation. This method assures us that by tuning the hyperparameters of our model, these will help to avoid overfitting the models. Several metrics can be used to compare the results, and for evaluating the task as a regressor, we use root mean squared error and *R*^2^. For the classification task, we compare area under the curve and *F*_1_-scores.

#### Question 2

##### Overview

What are the key predictive features that exhibit strong association with DASS21 responses from participants?

We aim to perform a feature selection process to identify the variables with the strongest association with the result of DASS21. This step involves selecting a subset of the most relevant features from a large set of potential predictors. It aims to simplify the model and reduce the risk of overfitting. Our data set will contain 3 different variable types: audio, text, and numeric variables. We will use other methods for each variable type to extract features and select the most significant ones.

##### Audio Features

In the case of audio features, this step will involve feature extraction techniques such as mel-frequency cepstral coefficients and discrete wavelet transform to extract the applicable features from the recordings. These techniques will help us transform the audio signal into a more meaningful representation that can be used as input for our machine learning model.

##### Text Features

In addition to extracting the audio signal, we aim to extract the transcript from the audio recordings and identify relevant semantic text features using word embeddings. The idea behind using word embeddings is to group similar words in a high-dimensional space, where words with similar meanings and emotional tones are close to each other. Our approach is to input the word vectors as sentences to the machine learning model to classify the emotional tone of the individuals’ responses to the open questions.

##### Numeric Features

We will use wrapper methods to select the most relevant features in our numeric attributes subset, such as recursive feature elimination. This method will evaluate the performance of our machine learning model with different subsets of features by considering the interaction between the distinct variables and their relationship with the target variable.

Finally, after extracting meaningful representations of the audio and text columns in our data set and compressing the numeric features, we will use dimensional reduction techniques such as principal component analysis to reduce the number of features further, while preserving the most critical information, allowing us to visualize the data in 2 or 3 dimensions.

#### Question 3

To what extent does personalized machine learning demonstrate superior predictive efficacy in mental health compared with traditional, one-size-fits-all models in the context of DASS21 responses?

Personalized machine learning aims to fill the gap of one-size-fits-all models when assessing mental health disorders in people. The collected features could have significant variability among individuals with equal DASS21 assessment. Hence, we aim to supply individual machine learning models trained with personal data to provide personal activity and a baseline to assess whether personalized models outperform cohort-trained models.

We should consider careful preprocessing to clean the data to train a machine learning model. First, we need to consider the presence of outliers. Outliers could bias or influence the decision of our trained algorithms. Hence, applying Mahalanobis distance to our numerical attributes obtained from the activity tracker can aid us in identifying abnormal points of data and treating them. We could cap the score to treat the outliers at a particular value above the 90th or below the 10th percentile. The imbalanced data set is another issue while applying machine learning for classification tasks. If the number of instances where one class label is significantly lower than the others, it could bias the model toward the majority class. Therefore, we should consider oversampling or undersampling techniques to remove this bias. Finally, we will consider removing background noise for voice analysis.

For personalized modeling, we should consider using multitasking learning techniques. These techniques leverage data from across the population with deep neural networks and hierarchical Bayesian models [36]. We could also consider implementing ensemble learning, such as random forest and gradient boosting, by training the models with the different data types for each person and using a voting regressor performance to select the most accurate model. The more data we obtain through time from the individual, the more the model will capture the patterns of the individual by retraining the models to adjust to the new data.

After training the models with the leave-one-out technique, we will compare the performance of the regression or classification models to draw our conclusions.

## Results

The data collection process started in June 2022. So far, 67% (29/43) of the participants have completed the study protocol, and 33% (14/43) are in the process. The study results will be available at the end of 2023 and published in scientific journals.

## Discussion

### Expected Findings

This study aims to describe the data collection experiment to capture the mood and activity of students in Canada and Mexico. This data set will consist of longitudinal data from each participant, allowing us to observe our participants’ behavior extensively. Each data set entry is labeled with the DASS21 questionnaire, enabling us to apply machine learning algorithms for prediction or classification. The data set includes physiological and activity data from some popular fitness trackers. This allows us to identify a subset of the common underlying data from these trackers. In addition, as we recruit English-speaking and Spanish-speaking participants, the data set will include 2 significant languages, allowing for rich analysis and comparison.

Overall, the high-level objective of this study aims to advance state-of-the-art personalized machine learning for mental health; generate a valuable data set that could be used to predict DASS21 results by applying algorithms of machine learning; and finally, deploy a framework that detects the onset of depression, anxiety, and stress to alert the user and prompt them to consult a professional. Moreover, we aim to compare traditional, cohort, machine learning methods against personalized methods and propose a methodology to detect multiple mood disorders not limited to depression, stress, and anxiety. Finally, we will compare the relative value of 2 speech data sources, free speech and simple text reading, for mood analysis.

### Limitations

When interpreting the results of the study, various limitations need to be taken into account. Our experiments are exclusively conducted on students within graduate and undergraduate environments. This decision was mainly based on the available resources and will narrow the conclusions of our study for this specific group.

Another limitation is the challenge regarding the participant’s adherence to the protocol and consistency of the data. The periodicity of the data might not be consistent for all the participants; thus, we require them to deliver 20 full days of complete data. However, we are planning to consider these gaps as part of the behavioral information that could lead us to the objective of personalized machine learning for DASS21 predictions.

Our methodology relies on the assumption that participants will provide true signals about their daily behavior and mental status. In principle, it is possible that they will remove their wearable devices during the study or that they may not respond truthfully to the DASS21 questionnaire. This is a potential flaw with all studies collecting and analyzing self-reported data, and it is almost impossible to eliminate. Nevertheless, we believe that our study protocol is designed in a manner that diminishes the likelihood of this problem. First, participation in our study is voluntary, and the monetary incentive was calculated to recognize the time that participants spend in following our protocol. Second, all data outside the consent form are anonymous and associated only with a nickname, which should alleviate any concerns that participants may have about being associated with the “stigma” of mental health challenges that might lead them to not answer truthfully. Furthermore, it is important to note that the DASS21 questionnaire has been extensively used and validated for Spanish-speaking and English-speaking users and adopted for depression, anxiety, and stress diagnosis.

### Conclusions

In this paper, we have described a protocol for collecting physiological data using wearable and mobile devices, which will be used in a subsequent study to predict depression using machine learning algorithms. The project is designed to collect physiological signals, such as heart rate variability, sleeping patterns, voice, and activity levels, which are reliable indicators of depression, anxiety, or stress. The noninvasive protocol consists of simple steps for the participants to follow. In this paper, we have outlined the procedures to ensure informed consent and data collection in a standardized and secure manner.

With the increasing prevalence of anxiety and depression disorders, this study has significant implications for developing a noninvasive and objective method for collecting and joining DASS21 results and data from wearables and mobile devices. The availability of the data set for the scientific community could motivate new and better ways for prompt detection of mood disorders. The proposed methods emphasize the importance of proper participant recruitment and data storage, collection, and processing. However, we recognize the limitations of our study, including the small sample size and need for further validations using more extensive and diverse populations, adherence of the participants to the study, and fidelity of the provided data. Further studies will be conducted to validate these findings and explore this technology’s potential for predicting other mental health disorders.

## Abbreviations

DASS21: Depression, Anxiety, and Stress Scale

## Data Availability

The data are not publicly available because the collection is ongoing and it has not been appropriately formatted. However, the data collection tools are available upon request from the corresponding author.
