# Correction: Using Wearable Devices and Speech Data for Personalized Machine Learning in Early Detection of Mental Disorders: Protocol for a Participatory Research Study

**DOI:** 10.2196/78287

**Published:** 2025-07-28

**Authors:** Ramon E Diaz-Ramos, Isabella Noriega, Luis A Trejo, Eleni Stroulia, Bo Cao

**Affiliations:** 1 Department of Computing Science University of Alberta Edmonton, AB Canada; 2 School of Engineering and Sciences Tecnologico de Monterrey Monterrey Mexico; 3 School of Engineering and Sciences Tecnologico de Monterrey Atizapan Mexico; 4 Department of Psychiatry University of Alberta Edmonton, AB Canada

In "Using Wearable Devices and Speech Data for Personalized Machine Learning in Early Detection of Mental Disorders: Protocol for a Participatory Research Study" (JMIR Res Protoc 2023;12:e48210), the authors noted an error.

The following references:

32. Chen I-M, Lin P-H, Wu V-C, Wu C-S, Shan J-C, Chang S-S, et al. Suicide deaths among patients with end-stage renal disease receiving dialysis: a population-based retrospective cohort study of 64,000 patients in Taiwan. J Affect Disord. Feb 2018;227:7-10. [doi: 10.1016/j.jad.2017.10.020] [Medline: 29045916]

33. Wang F, Wang Y, Tian Y, Zhang P, Chen J, Li J. Pattern recognition and prognostic analysis of longitudinal blood pressure records in hemodialysis treatment based on a convolutional neural network. J Biomed Inform. Oct 2019;98:103271. [FREE Full text] [doi: 10.1016/j.jbi.2019.103271] [Medline: 31454648]

Have been revised to 32 and 33:

32. Chen C, Liu C-C, Weng P-Y, Cheng Y. Mismatch Negativity to Threatening Voices Associated with Positive Symptoms in Schizophrenia. Front Hum Neurosci. 2016;10:362. [FREE Full text] [doi: 10.3389/fnhum.2016.00362] [Medline: 27471459]

33. Wang D, Ding Y, Zhao Q, Yang P, Tan S, Li Y. ECAPA-TDNN Based Depression Detection from Clinical Speech. In Interspeech. In Interspeech. 2022;10:3333-3337. [doi: 10.21437/Interspeech.2022-10051]

The correction will appear in the online version of the paper on the JMIR Publications website, together with the publication of this correction notice. Because this was made after submission to PubMed, PubMed Central, and other full-text repositories, the corrected article has also been resubmitted to those repositories.
